# Construction of a High-Density Genetic Map and Quantitative Trait Locus Mapping in the Sea Cucumber *Apostichopus japonicus*

**DOI:** 10.1038/srep14852

**Published:** 2015-10-06

**Authors:** Meilin Tian, Yangping Li, Jing Jing, Chuang Mu, Huixia Du, Jinzhuang Dou, Junxia Mao, Xue Li, Wenqian Jiao, Yangfan Wang, Xiaoli Hu, Shi Wang, Ruijia Wang, Zhenmin Bao

**Affiliations:** 1 Ministry of Education Key Laboratory of Marine Genetics and Breeding, College of Marine Life Sciences, Ocean University of China, 5 Yushan Road, Qingdao 266003, China

## Abstract

Genetic linkage maps are critical and indispensable tools in a wide range of genetic and genomic research. With the advancement of genotyping-by-sequencing (GBS) methods, the construction of a high-density and high-resolution linkage maps has become achievable in marine organisms lacking sufficient genomic resources, such as echinoderms. In this study, high-density, high-resolution genetic map was constructed for a sea cucumber species, *Apostichopus japonicus*, utilizing the 2b-restriction site-associated DNA (2b-RAD) method. A total of 7839 markers were anchored to the linkage map with the map coverage of 99.57%, to our knowledge, this is the highest marker density among echinoderm species. QTL mapping and association analysis consistently captured one growth-related QTL located in a 5 cM region of linkage group (LG) 5. An annotated candidate gene, retinoblastoma-binding protein 5 (*RbBP5*), which has been reported to be an important regulator of cell proliferation, was recognized in the QTL region. This linkage map represents a powerful tool for research involving both fine-scale QTL mapping and marker assisted selection (MAS), and will facilitate chromosome assignment and improve the whole-genome assembly of sea cucumber in the future.

Genetic linkage maps have become a critical and indispensable tool in an extensive range of genetic and genomic research^1^. A genetic map with sufficient density and resolution is one of the essential prerequisites for marker-assisted selection (MAS), comparative analysis of genomic synteny, fine mapping of quantitative trait loci (QTL), positioning of candidate genes and facilitating the chromosome assignment of whole-genome scaffolds. The resolution and density of a genetic linkage map primarily depends on the number of mapping individuals and mapping families, as well as that of candidate genetic markers and the corresponding accuracy of genotyping in the mapping families^2^. Therefore, it is understandable that the improvement of linkage map resolution is always accompanied by the technical innovation of marker development. Most of the first generation linkage maps for important aquatic species were constructed using both dominant and codominant markers, such as AFLP, RAPD and SSR markers, in tilapia^3^, channel catfish^4^, Pacific oyster^5^, and white shrimp^6^. However, these maps did not demonstrated a sufficient capability in studies addressing the fine mapping of QTLs or MAS due to a low density and the technical limitations of genetic markers^7^. With advances in molecular genetics, co-dominant markers including microsatellites (SSR) and single nucleotide polymorphisms (SNPs), began to be applied in the construction of linkage maps due to their high abundance and mapping potential^8^. The low efficiency and high cost of traditional strategies for marker development become the major obstacles that restricting the usage of these techniques in the generation of high-density genetic linkage maps^9^. Recent advances in next-generation sequencing technologies have enabled the identification of sufficient genetic molecular markers at a reasonable price, thereby promoting the development of several high-throughput SNP genotyping methods, particularly the restriction site-associated DNA (RAD) technique^10^ and its derivative methods. Among such techniques, 2b-RAD^11^, a representative optimized method that integrates the advantages of simpler library preparation, even and variable genome coverage, and a flexible genotyping platform, has been successfully applied to aquaculture species with large genomes^12^,^13^,^14^,^15^.

Echinoderms are the second largest group of deuterostomes, and they are the largest phylum without any freshwater or terrestrial (land-based) species^16^. Echinoderms are important both biologically and evolutionarily^15^. Biologically, they constitute one of the groups that is most abundantly distributed in the biotic desert of the deep sea, in addition to shallower ocean regions^17^. The two remarkable traits of echinoderms, tissue regeneration and aestivation, have become research hotspots attracting increasing attention from biologists^18^. Evolutionarily, it is believed that the radiation of echinoderms was responsible for the Mesozoic Marine Revolution^19^, and ossified skeletons of echinoderms are considered to be the major contributors to many limestone formations, providing valuable resources for tracking the ancient environment^20^. In addition, echinoderms comprise several species that are commercially important food resources around the world, with production of over 177 thousand tons being reported in 2012 and a total value of up to 622 million dollars^21^. Hence, genetic inheritances underlying economically important traits, such as growth and reproduction, have been central tasks in echinoderms genetic and breeding studies on echinoderms. Great progress has recently been achieved using RNA-Seq technology for the large-scale identification of candidate genes and SNPs in the echinoderms transcriptome^22^. However, the development of linkage maps has stalled in the whole echinoderm phylum. The only linkage maps constructed thus far are for *Strongylocentrotus nudus*, *Strongylocentrotus intermedius* and *Apostichopus japonicus*, using AFLP, SSR and SNP markers^23^,^24^. The density and resolution of these maps is generally low (mostly 7.0–17.1 cM), thus limiting their application in QTL related studies.

The sea cucumber *Apostichopus japonicus*, a representative echinoderm species, has become one of the most dominantly and economically important aquaculture species in the Western Pacific Ocean along the coasts of China, Japan, Korea and Russia, with considerable edible and medicinal value^25^. Sea cucumber breeding was first studied in China in 1954, and great progress was subsequently made in the breeding and larval rearing of sea cucumbers in the late 1990s^26^. In 2012, the farming area of *A. japonicus* in China had reached 153 thousand square hectometers, and production was more than 109 thousand tons^27^. The great expansion of large-scale sea cucumber farming has led to an increasing demand to promote aquaculture traits using MAS. Significant progress has been made in sea cucumber genomic research, particularly in the development of genetic and genomic resource including various types of polymorphic markers^22^, two low density linkage maps^23^,^28^ and great numbers of transcriptome contigs^29^. In this study, we describe the construction of a genetic linkage map with over seven thousand markers for *A. japonicus*, which is, to our knowledge, the first high-density, high-resolution genetic map to be reported for echinoderms. One major growth-related QTL interval was identified utilizing this map. This linkage map represents a powerful tool for research involving both fine-scale QTL mapping and MAS and will facilitate chromosome assignment for sea cucumber whole-genome assembly in the future.

## Results

### 2b-RAD sequencing and selection of markers

Single-end sequencing of 2b-RAD libraries was conducted using the Illumina HiSeq2000 platform on 100 progenies and their parents. A total of over 387 million reads were generated, including 29 million reads from the male parent, 37 million reads from the female parent, and 321 million reads from the progenies (3.2 million reads per progeny). The statistical sequencing depth corresponded to 81× in the male, 104× in the female and 17× in the 100 progenies. After quality trimming, 85% of the reads from the male parent, 98% of the reads from the female parent, and 91% of the reads from the progenies were carried forward for analysis (Table 1 and Additional File A1). The raw read data were archived at the NCBI Sequence Read Archive (SRA) under Accession Number PRJNA274721.

The genotyping procedure was performed using RADtyping v1.0^30^. The result showed that clustering of parental reads generated 186,692 representative reference tags, including 125,616 codominant tags and 61,076 dominant tags, 7769 low quality tags were removed according to their sequencing depth. In this study, we defined “dominant tags” and “dominant markers” as 2b-RAD tags that appear in only one parent. And “codominant tags” was defined as 2b-RAD tags that appear in both parents with allelic variations, while “codominant markers” represented those SNPs that detected in “codominant tags” (Supplementary Figure F1). Utilizing the constructed reference, 47,049 codominant markers and 57,722 dominant markers were detected. After initial genotyping, a total of 32,012 markers that showed polymorphism in at least one parent and 3 progenies were retained, including 20,169 codominant markers and 11,843 dominant markers (Table 2). Then the mendelian fit test and genotyping percentage were performed for further trimming. In total 11,306 markers that fit mendelian ratios (P ≥ 0.05) and high genotyping rate (got available genotype over 80 progenies) were used to construct sex specific map. Ultimately, 6311 and 5517 markers were used for female-specific and male-specific linkage map construction, respectively (Table 2 and Additional File A2).

### Linkage mapping and analysis of recombination rates

Linkage maps were first constructed separately for the male parent and female parent using Joinmap 4.0^31^ (LOD = 5.0). In total 6311 and 5517 markers were used to construct female and male map separately, after linkage mapping, 2302 and 1525 markers were eliminated. For both sex-specific maps, 22 linkage groups (LGs) were obtained (Supplementary Figure F2 and F3), which were consistent with the haploid chromosome number of *A. japonicus*^32^. The female genetic map consisted of 4009 markers mapped on 2908 distinct positions spanned 3728.9 cM, with an average marker interval of 0.93 cM, ranging from 0.72 in LG5 to 1.31 in LG14, while the male genetic map contained 3992 markers on 2138 distinct positions, with a much shorter genetic length (1824.3 cM) and shorter average marker interval (0.46 cM) (Table 3). The marker interval of the male genetic map ranged from 0.26 in LG8 to 0.99 in LG7 (Table 3). Overall, the length of the female genetic map was 1904.6 cM longer than the male genetic map, with an average female-to-male length ratio of 2.11:1. This ratio varied by linkage groups, ranging from 1.21:1 in LG1 to 3.45:1 in LG12 (Table 3).

**Figure 1:**
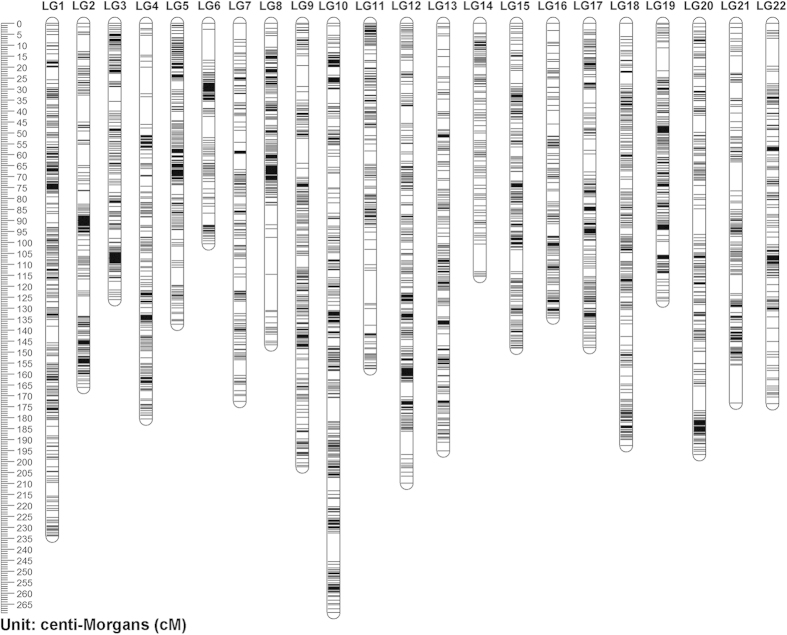
Recombination rate of shared markers between sex-specific maps and consensus map. This diagram was constructed using the recombination rate of 162 shared markers shared by sex-specific maps and consensus map. The left Y-axis represents shared marker interval in female map and right Y-axis represents the shared marker interval in male map, the X-axis stands for shared marker interval in consensus map. The blue dots stand for shared marker interval ratio between female map and consensus map (F:C ratio), while the red dots represent shared marker interval ratio between male map and consensus map (M:C ratio). *F:C ratio represents the recombination rate correlation of shared markers between female map and consensus map; M:C ratio stands for the recombination rate ratio between male map and consensus map.

Considering the relationship between interval distance and recombination, this result demonstrated significantly higher recombination rates in a majority of the linkage groups of the female compared with the male (t-test, p < 0.0001). Significantly different recombination rates (female-to-male recombination rate ratio >2.5) between the female and male maps were observed in LG2, LG3, LG4, LG5, LG8, LG17, and LG19 (Table 3). Across all of these linkage groups, the female:male recombination ratio ranges from 0.66:1 in LG 20 to 4.86:1 in LG 19 (Table 3). In total, 162 markers were shared between the two sexes, and the average recombination rates of these markers in each linkage group were not the same as for the sex-specific markers (Fig. 1). The female:male ratios for the recombination rates of shared markers for each linkage group ranged from 0.67:1 in LG5 to 4.32:1 in LG12. The highest recombination rate ratios (greater than 2.5) for markers shared between the sexes were observed in LG8 4.32:1, 2.61:1 in LG12 and 3.64:1 in LG17 (Supplementary Table T1), whereas the corresponding average recombination ratios for the sex-specific maps were 3.28:1, 1.44:1 and 2.58:1 (Table 3). While the recombination rates of shared markers in LG2 and LG14 were similar between the sexes (0.92:1, 1.06:1, respectively), various recombination rates were observed for the sex-specific maps (Supplementary Table T1).

**Figure 2:**
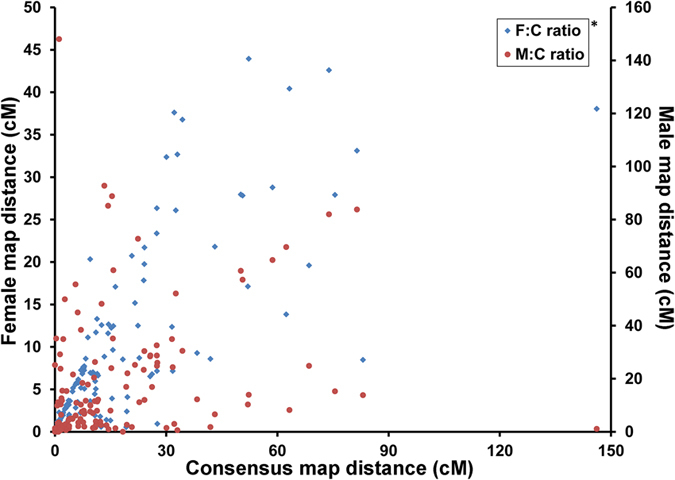
The high-density consensus linkage map of *A. japonicus*. The consensus map which contained 7839 markers in 22 linkage groups was constructed through combing the male and female linkage maps (Supplementary Figure F2 and F3).

The consensus linkage map for *A. japonicus* was constructed after integration of the two sex-specific maps using 162 heterozygous markers from both parents. This consensus map comprised 7839 markers representing 4884 distinct positions spanning 3706.6 cM, with an average resolution of 0.47 cM (Fig. 2). The size of each linkage group ranged from 100.6 cM to 268.8 cM, and the number of loci varied from 174 to 618 per linkage group, with an average of 168.5 cM and 356 loci per group (Table 4). According to the constructed linkage maps, the patterns of marker distribution across chromosomes can be examined (Supplementary Figure 2, 3 and Fig. 2). Clustered markers were commonly found in regions around the center of linkage groups and less frequently observed around terminal regions. This result was consistent with observations in various genetic maps of aquatic species previously^33^,^34^.

### Map length, map coverage and map validation

According to the two different algorithms, the expected map length was estimated to be 3716.9 cM (G_e1_) and 3728.5 cM (G_e2_), with an average expected map length of 3722.7 cM (G_e_) being obtained. The map coverage of this consensus map was 99.57% based on the expected map lengths according to the average length calculated from the following formulas: 1) G_e1_ = G_of_ + 2s, in which G_of_ represents the observed length of each linkage group, and s represents the average interval of the whole map; 2) G_e2_ = L × (m + 1)/(m − 1), where L represents the observed length of the linkage groups, and m is the number of markers for the corresponding group^35^.

To confirm the reliability of the genotyping information used in linkage map construction, we performed re-sequencing of both parents and randomly selected 10 progenies. The results showed that the genotyping consistency was as high as 96.9%, 95.0% and 94.06% in the male parent, female parent and offspring, respectively (Supplementary Table T2). The validation results provided solid evidence of an acceptable quality of the high-density linkage map.

### QTL mapping and association analyses of growth trait

**Figure 3:**
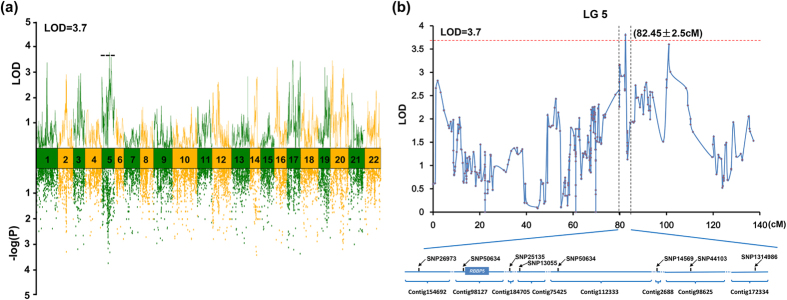
Growth-related QTL mapping and association analysis in *A. japonicus* among all linkage groups (a) and the candidate region of the major QTL in LG5 (b). The dashed line indicates the genome-wide significance threshold where LOD = 3.7. The vertical dashed line stands for the growth trait related QTL window (82.45 ± 2.5 cM).

Multiple QTL model (MQM mapping) in MapQTL^36^ package was used for analyzing the body weight information of 100 progenies. After QTL mapping, a linkage region on LG5 was detected to pass the corresponding LOD threshold (LOD ≥ 3.7) of the corresponding permutation test and was considered as the major QTL in this study. This QTL (SNP37318) was situated at 79.95–84.95 cM in LG5, explaining 11.8% of the phenotypic variation in body weight observed in *A. japonicus*. In total 7839 markers on the linkage map were performed for association analysis using TASSEL 5.0^37^ and the P-value was adjusted to WFDR-P by WFDR (Weighted False Discovery Rate)^38^ with cutoff 0.1 (Additional File A3). Association analysis showed similar distribution patterns to the QTL mapping analysis across all linkage groups (Fig. 3a), supporting the results of the QTL analyses overall. The nine closest loci (Fig. 3b) located near the growth-related QTL genomic window ( ± 2.5 cM) were defined as the candidate locus relevant to the grow traits of *A. japonicus*. Eight of these nine loci could be mapped onto eight scaffolds of the *A. japonicus* draft genome (unpublished data), among which one 47.2 kb scaffold (Contig98127) had available gene annotation information (Table 5 and Fig. 3b). One annotated gene, retinoblastoma-binding protein 5 (*RbBP5*), which has been reported to be an important regulator in cell proliferation^39^, was 190 bp downstream of the candidate markers (SNP50634).

## Discussion

In the present work, we constructed the first high-density genetic map reported for echinoderms, in the sea cucumber aquaculture species *A. japonicas*. This map possesses the highest marker density among all of the genetic linkage maps constructed for any echinoderm species. Taking advantage of 2b-RAD technology, we were able to cost-effectively genotype 32,012 polymorphic markers in 102 sea cucumbers (two parents and 100 offspring) from one full-sibling families (Table 2). Such a high-density linkage map is expected to be a valuable resource for genomic analyses and fine-scale QTL mapping in the sea cucumber *A. japonicus*.

“High density” refers to a compact marker interval (<2 cM), high map coverage and even marker distribution in a genetic linkage map^40^. This term usually indicates that tens of thousands candidate polymorphic markers are obtained for species with large (>500 MB) and complex genomes^41^. Therefore, a cost-effective sequencing and genotyping plan which can balance the marker quantity, sequencing coverage, sequencing cost and genotyping accuracy, is the foremost guaranteed for the construction of large-scale linkage maps in the non-model invertebrate species without the whole genome reference, such as sea cucumber, *A. japonicus*^30^. An optimized 2b-RAD sequencing plan^11^ was applied in this study, which can easily provide the sequencing coverage required for a given sequencing depth, considering the genome size (880 MB) reported for sea cucumber^42^. As other next-generation sequencing-based technology, 2b-RAD possess the following advantages: 1) lower sequencing cost due to the sequencing of a subset of BsaXI sites derived from RR libraries, rather than a whole genome; 2) an adjustable marker density due to utilizing selective adaptors; and 3) a relatively even marker distribution, benefiting from the ubiquitous restriction sites in the genome^11^.

High levels of polymorphism and heterozygosity have come to be considered universal hallmarks of most invertebrate genomes, including those of several mollusks, chordates and echinoderms^43^,^44^,^45^. In sea urchin, the existence of over 2% genomic polymorphism markedly decreased the performance of genome assembly compared with other vertebrates^44^. Not coincidentally, a high degree of genome polymorphism and heterozygosity have significantly increased the difficulty of the assembly process in the ongoing starfish genome project (EchinoBase, http://www.echinobase.org/Echinobase/PmBase). Conversely, the level of genomic polymorphism can be inferred from the development of polymorphic markers, such as AFLPs, SSRs and SNPs^46^. In this study, 32,012 polymorphic markers were identified in the evaluated full-sib mapping family after 2b-RAD genotyping (Table 2). This number was 4.29 times higher than that obtained in a previous 2b-RAD study in *Chlamys farreri*^12^. One technical difference between these two projects is the utilization of two types of selective adaptors (5′-NNT-3′ and 5′-NNA-3′) in this study, while one type of selective adaptor (5′-NNT-3′ ) were used in *Chlamys farreri*^12^. The selective adaptor used in this study means one type of adaptor with a single selective base at 3′ end. Theoretically, compared with using only one type of selective adaptor, the extra type of selective adaptor will quadruple the restriction sites in the genome leading to the proportional expansion of the polymorphic markers (Supplementary Figure F4). However, after the consideration on the smaller genome size of *A. japonicu* (~880 MB)^42^ than *C. farreri* (~1.24 GB)^47^, the total amount of polymorphic markers identified from sea cucumber was still relatively high. The abundance of polymorphic markers implied the high polymorphisms, complexity, and heterozygosity features of *A. japonicus* genome, which required special attentions and considerations in the future whole genome assembly.

The construction of genetic linkage maps for echinoderms is still in its infancy. To date, only a few genetic linkage maps have been constructed for echinoderm species^23^,^24^,^28^. However, these maps were generally constructed at a low density, using several hundred molecular markers at most. Even for the sea cucumber *A. japonicus*, for which the greatest number of linkage maps are available, the mapping density was still above 7.0 cM^23^,^28^. An insufficient map density has been one of the barriers limiting the development of both genomics and genetics. In this study, we adopted the double pseudo-testcross strategy to construct the first high-density SNP genetic linkage map for *A. japonicus* by taking advantage of parental heterozygosity^42^,^48^. The final consensus linkage map contained 7839 markers with a resolution of 0.47 cM (Table 4), surpassing the resolution of not only the previous genetic maps for *A. japonicus* but also that of linkage maps reported for other echinoderms (7.0–17.1 cM)^23^,^24^,^28^, and is even competitive with other genetic linkage maps constructed through 2b-RAD (0.39–0.41 cM)^12^,^15^.

Another common issue of RAD-based method is allele/locus dropout problems in genotyping for dominant tag. Here we utilized a new analytical tool, RADtyping, which provides a statistical algorithm to avoid sampling error for accurate *de novo* dominant genotyping in mapping population^30^. Specifically, we first removed the low-quality sites that are not supported by parental reads in sufficient depth (i.e. the requirements *d*_pl_ < *l*_pl_ and *d*_p2_ < *l*_p2_), where the thresholds are determined by:


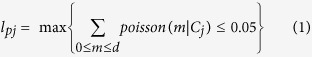


*C*_j_ is the mean sequence depth of the *j* th parent (*j* = 1, 2). Then, the low-coverage sites in the progenies with *d*_pro_ less than *l*_pro_ were removed, where *d*_pro_ is calculated for each site by summarizing all progenies having reads derived from that site, and the threshold *l*_pro_ is determined for each site using this formula again with parameter *C*_j_ being the average depth of all progenies. At the stage of the dominant genotyping, supposing that the cluster depth of the *i*th site for the *j* progeny is *d*_ij_, this site is called as “presence” if *d*_ij_ > *l*_d_, “absence” if *d*_ij_ = 0, and unknown if *d*_ij_ ranges from 0 to *l*_d_, where the threshold *l*_d_ is determined using formula with *C* being the mean sequencing depth of the *i*th site. The performance of above statistical algorithm has been thoroughly evaluated using both simulated and real sequencing datasets previously with genotyping accuracy higher than 97.6% for all cases^30^.

The sea cucumber *Apostichopus japonicus* is one of the most important species of commercial echinoderms in aquaculture. Growth traits are of particular interest to *A. japonicus* researchers due to their high commercial significance in the sea cucumber culture industry. QTL mapping represents an efficient approach for identifying the genetic loci underlying these traits, to allow marker-assisted selection to be applied in genetic breeding. One major QTL related to total body weight was mapped to a 79.95–84.95 cM region in LG5 in this study (Fig. 3b). Thus, this region was considered to be a candidate genomic region involved in controlling the growth of *A. japonicus*. Although the gapped draft genome assembly (unpublished data) may limit QTL candidate gene mining, a growth-related gene, retinoblastoma-binding protein 5 (*RbBP5*), was recognized within the loose window close to this QTL (Fig. 3b). *RbBP5* encodes a ubiquitously expressed nuclear protein that belongs to a highly conserved subfamily of WD repeat proteins. This protein binds directly to retinoblastoma protein, which regulates cell proliferation^49^. *RbBP5* has also been reported to show the ability to regulate *HOXA9* and *HOXC8* to control cell growth and cell differentiation^50^. Knockdown of *RbBP5* led to a reduction of the expression of the HOX gene family members *HOXA9* and *HOXC8*, which play a crucial role in embryonic and fetal development^51^. Our QTL analysis provided a candidate gene that could be exploited in the future selective breeding of *A. japonicus* with the aim of improving sea cucumber aquaculture production. Further studies are necessary to explore the detailed regulatory mechanisms of *RbBP5* involved in cell proliferation and growth in sea cucumber with more families and populations.

## Materials and Methods

### Resource family and DNA extraction

A total of nine full-sib families were established in April 2009 through mating between a pair of common male and nine different females. The parents were selected from wild population in Penglai, Shandong province. And the families were constructed in an aquatic breeding farm in the city of Weihai, Shandong province of China. Body weights were measured and recorded for all families at the age of one year. One of these families exhibiting high within-family variation in growth traits was chosen for linkage and QTL analysis. A total of 100 one-year-old offspring were randomly selected from this family, and mixed tissues from all specimens, including parents and offspring, were collected and stored in 70% ethanol for DNA extraction. Genomic DNA was prepared following previously described traditional phenol-trichloromethane DNA extraction procedures^52^. The body tissues of experimental individuals were firstly cut into pieces and digested in 2% CTAB by holding at 56 °C for 1 hour. After digestion process, cycles of phenol-trichloromethane extraction were conducted to eliminate protein. Then DNA was precipitated in ethanol and washed by 70% ethanol for 2-3 times to eliminate micro molecule. RNA digestion was conducted by adding proper quantities of RNase and holding at 37 °C for 30 minutes. After concentration and integrity examination, DNA samples could be used for further experiments. All of the procedures involved in the handling and treatment of sea cucumbers during this study were approved by the Ocean University of China Institutional Animal Care and Use Committee (OUC-IACUC) prior to the initiation of the study. And all experiments and relevant methods were carried out in accordance with approved guidelines and regulations of OUC-IACUC.

### 2b-RAD sequencing

Before the experiment was carried out, a digital-enzyme-cut analysis was conducted on the draft genome assembly to confirm the sufficient amount of restriction sites. According to the analysis of the restriction sites, the two types of selective adaptors (5′-NNT-3′ and 5′-NNA-3′) with a selective base on the 3′end were finally decided to generate reasonable quantities of tags used for further analysis. For the construction of 2b-RAD libraries, two parents and 100 offspring were used, following the standard protocol^11^. Briefly, DNA was digested with BsaXI at 37 °C for 4 hours and then ligated to adapters at 4 °C for 16 hours to provide the complementary sequences for primers. For the parents, standard BsaXI libraries were constructed, while for progenies, reduced representation (RR) libraries were constructed using adaptors with 5′-NNT-3′ and 5′-NNA-3′ overhangs to target a subset of BsaXI fragments in *A. japonicus*. Pre-amplification reactions were performed using BsaXI primers to enrich the DNA fragments ligated to adaptors (14 cycles, annealing temperature 60 °C). The amplification products were purified via retrieval from 8% polyacrylamide gels. The 2^nd^ amplification proceeded using a BsaXI-barcode primer with two selective bases and the pre-amplification products as a template to distinguish libraries for different individuals (7 cycles, annealing temperature 60 °C). The 2b-RAD libraries were then pooled for single-end sequencing using the Illumina HiSeq2000 system.

### Preprocessing and genotyping of sequence data

Raw reads were processed for initial trimming using a homemade Perl script. Adaptor sequences, unreliable reads (no restriction sites or ‘N’ at the end of reads), long homopolymeric regions (>10 bp), low-quality sequences (>5 positions with a quality <0) and mitochondrial origin were removed^30^. RADtyping v1.0 (http://www2.ouc.edu.cn/mollusk/detailen.asp?Id = 727) was employed in the following genotyping procedure due to its acceptable performance for 2b-RAD data^12^. Sequences from parents with BsaXI tags were first clustered under default parameters (−m 3, −M 2). The clusters with low coverage (<8) or excessive coverage (>3000) were removed to avoid the interference of sequencing errors and repetitive elements. A total of 7769 clusters were removed and the remaining clusters were used to construct the reference sequences and identify the markers. The clusters were further classified into codominant tags and dominant tags for subsequent codominant markers and dominant markers genotyping. Sequencing reads of offspring were mapped on the constructed high-quality reference. For codominant markers with sufficient reads coverage (6–500), we used iML algorithm^53^ to estimate their homozygous or heterozygous. On the other side, dominant markers were recorded as “presence” or “absence” by RADtyping to reflect its existence. Markers that showed polymorphism in at least one parents and 3 progenies were kept for further trimming. Then the markers that fit Mendelian ratios (P ≥ 0.05) and sufficient genotype rate (got available genotype over 80 progenies) were used to construct sex specific map.

### Linkage map construction and map validation

The dominant markers that segregated at a 1:1 ratio in the map family were obtained and categorized as lm × ll (markers from the male parent) or nn × np (markers from the female parent). Markers present in both parents that segregated at a 1:2:1 ratio were also retrieved and were defined as hk × hk (markers in both parents). Prior to makers grouping, the markers were tested for goodness-of-fit based on the expected Mendelian ratios through chi-square tests to eliminate markers that significantly deviated from the expected ratios (p-value ≤ 0.05). Male- and female-specific linkage maps were then grouped and constructed using the qualified markers with JoinMap 4.0^31^. The genetic positions of the markers were determined using an LOD score cut-off of 6.0, and the recombination frequencies were converted into map distances in centi-Morgans (cM) through the Kosambi mapping function. The consensus map was then established by integrating sex-specific maps through shared markers using MergeMap^54^, map weight was set to 1.0 for both sex-specific maps. The visualized linkage maps were subsequently drawn using MapChart 2.2^55^.

To confirm the reliability of the genotyping information used in linkage map construction, re-sequencing was employed in two parents and 10 progenies using the HiSeq2000 platform as full technical replicates to confirm the accuracy of genotyping (supplementary table T2).

### Estimation of expected map length and map coverage

Once the linkage map was constructed, the expected map length (G_e)_ of *A. japonicus* was estimated in two different ways. Expected map lengths was calculated by the following formulas: 1) G_e1_ = G_of_ + 2s, in which G_of_ represents the observed length of each linkage group, and s represents the average interval of the whole map; 2) G_e2_ = L × (m + 1)/(m − 1), where L represents the observed length of the linkage groups, and m is the number of markers for the corresponding group^35^. Briefly, two different methods were employed to estimate expected map length G_e1_ and G_e2_^35^, and the average of these two indexes was used as the expected map length. The observed map coverage (C_oa_) for *A. japonicus* was determined by calculating the G_oa_/G_e_ value, where G_oa_ was the observed total genetic map length.

### QTL mapping of growth traits

QTL mapping analysis was performed for the body weight of *A. japonicus* using MapQTL^36^. The LOD scores were first analyzed using the multiple QTL model (MQM mapping), after which genome-wide and chromosome-wide LOD significance thresholds at the 95% level were determined with a 1000-permutation test for body weights, and QTLs with LOD scores greater than the LOD threshold at 95% were declared significant. Once a QTL was detected, the confidence interval was calculated followed the protocol of Li’s method^56^.

Association analyses between genotypes and growth traits were conducted using TASSEL 5.0^37^, which performs a least squares fixed effects linear model of quantitative trait on the genotype. Integrating the result of QTL mapping and association analysis, when a QTL was captured for significant correlation with body weight, sequencing tags were extracted. Then both homolog based analysis (BLAST and gene-wise alignment) and de novo gene prediction (Augustus^57^) was performed on the corresponding genomic scaffolds.

## Additional Information

**How to cite this article**: Tian, M. *et al*. Construction of a High-Density Genetic Map and Quantitative Trait Locus Mapping in the Sea Cucumber *Apostichopus japonicus*. *Sci. Rep*. **5**, 14852; doi: 10.1038/srep14852 (2015).

## Supplementary Material

Supplementary Information

Supplementary Table S1

Supplementary Table S2

Supplementary Table S3

## Figures and Tables

**Table 1:** Summary of data filtering of *A. japonicas*.

	Raw-reads	High-quality reads	Sequencing depth
**Male**	29,077,897	24,588,767(85%)	81×
**Female**	37,471,929	36,811,645(98%)	104×
**Progeny**	3,210,181	2,838,723(91%)	17×

**Table 2:** Markers selected for linkage mapping.

	Number
Markers detected in parents	104,771
Markers polymorphic and with high genotype percentage	32,012
Markers used for linkage mapping after Mendelian ratio trimming	11,306
Markers used for female linkage mapping	6311
Markers used for male linkage mapping	5517

**Table 3:** Summary of sex-specific linkage maps of *A. japonicas*.

LG	Female-specific map					Male-specific map					F:M ratio	
	Mapped markers	Distinct positions	Genetic length (cM)	Marker interval (cM)	Average recombination rate	Mapped markers	Distinct positions	Genetic length (cM)	Marker interval (cM)	Average recombination rate	Map length ratio	Average recombination rate ratio
**1**	233	178	171.8	0.74	0.005	288	176	142.4	0.49	0.007	1.21	1.49
**2**	154	89	115.4	0.75	0.039	263	120	89.6	0.34	0.136	1.29	3.54
**3**	130	109	136.6	1.05	0.072	188	115	82.8	0.44	0.214	1.65	2.98
**4**	169	125	179.9	1.06	0.065	221	95	84.6	0.38	0.175	2.13	2.70
**5**	205	135	148.1	0.72	0.071	213	95	78.6	0.37	0.151	1.88	2.13
**6**	95	67	99.8	1.05	0.126	127	63	78.4	0.62	0.339	1.27	2.70
**7**	184	140	168.5	0.92	0.246	68	40	67.7	0.99	0.183	2.49	0.74
**8**	193	110	148.3	0.77	0.056	312	106	82.5	0.26	0.182	1.80	3.28
**9**	223	159	204.4	0.92	0.083	219	120	79.7	0.36	0.167	2.56	2.01
**10**	355	251	271.1	0.76	0.009	276	129	114.9	0.42	0.012	2.36	1.33
**11**	158	117	170.8	1.08	0.031	107	73	75.9	0.71	0.039	2.25	1.24
**12**	280	212	218.7	0.78	0.021	193	95	63.3	0.33	0.030	3.45	1.44
**13**	186	146	211.5	1.14	0.027	175	102	78.1	0.45	0.056	2.71	2.06
**14**	86	62	112.4	1.31	0.059	93	63	66.1	0.71	0.135	1.70	2.31
**15**	203	143	159.3	0.78	0.034	184	107	78.4	0.43	0.065	2.03	1.92
**16**	142	95	162.6	1.15	0.057	120	67	60.8	0.51	0.104	2.67	1.83
**17**	179	129	156.6	0.87	0.035	216	101	81.8	0.38	0.091	1.91	2.58
**18**	186	157	210.7	1.13	0.047	181	116	80.0	0.44	0.099	2.63	2.13
**19**	89	76	138.1	1.55	0.046	201	147	83.7	0.42	0.224	1.65	4.86
**20**	260	185	201.0	0.77	0.134	81	54	61.7	0.76	0.088	3.26	0.66
**21**	149	114	163.0	1.09	0.088	134	76	95.0	0.71	0.165	1.72	1.89
**22**	150	109	180.3	1.20	0.096	132	78	98.3	0.74	0.176	1.83	1.83
**Total**	4009	2908	3728.9	0.93	0.066	3992	2138	1824.3	0.46	0.129	2.11	2.17

**Table 4:** Summary of the consensus linkage map of *A. japonicas*

Linkage group	Consensus map						
	Mapped markers	Female-specific makers	Male-specific markers	Shared makers	Distinct positions	Genetic length (cM)	Marker interval (cM)
**1**	506	202	264	40	339	234.0	0.46
**2**	416	151	257	8	208	166.1	0.40
**3**	309	110	171	28	215	126.1	0.41
**4**	383	155	212	16	213	180.5	0.47
**5**	415	193	208	14	227	137.3	0.33
**6**	219	86	120	13	127	100.6	0.46
**7**	245	174	59	12	173	172.5	0.70
**8**	495	181	299	15	206	146.7	0.29
**9**	437	212	211	14	274	202.4	0.46
**10**	618	332	257	29	367	268.8	0.43
**11**	257	139	97	21	182	157.7	0.61
**12**	462	263	180	19	296	209.8	0.45
**13**	348	162	160	26	235	195.1	0.56
**14**	174	78	83	13	120	115.5	0.66
**15**	374	183	166	25	237	148.2	0.40
**16**	257	135	114	8	157	134.4	0.52
**17**	391	173	201	17	226	148.0	0.38
**18**	361	175	174	12	267	192.7	0.53
**19**	285	79	186	20	218	126.7	0.44
**20**	334	244	73	17	232	196.8	0.59
**21**	280	142	129	9	187	173.2	0.62
**22**	273	134	122	17	178	173.6	0.63
**Total**	7839	3703	3743	393	4884	3706.6	0.47

**Table 5:** Information of growth-related QTL window in LG5.

**Maker**	**Genetic distance (cM)**	**Scaffold ID**	**Scaffold length (bp)**	**Annotation**
**SNP2336306**	80.01	–	–	–
**SNP26973**	80.61	Contig154692	44,607	–
**SNP50634**	82.04	Contig98127	47,215	*RBBP5*
**SNP25135**	82.19	Contig184705	2493	–
**SNP37318**	82.45	–	–	–
**SNP12502**	83.11	–	–	–
**SNP13055**	83.26	Contig75425	26,055	–
**SNP14569**	83.26	Contig112333	98,411	–
**SNP3266**	83.26	Contig2688	3525	–
**SNP44103**	83.26	Contig98625	40,991	–
**SNP1314986**	83.33	Contig172334	26,407	–
**SNP35302**	84.42	–	–	–
