# Supplementary material for: Construction of a High-Density Genetic Map and Quantitative Trait Locus Mapping in the Sea Cucumber Apostichopus japonicus
Source: Sci Rep. 2015 Oct 6;5:14852. doi: 10.1038/srep14852 (PMC4594301; doi:10.1038/srep14852)
Supplement: Supplementary Information [file srep14852-s1.pdf]

Construction of a High-Density Genetic Map and Quantitative Trait Locus Mapping in Sea Cucumber, *Apostichopus japonicus*

Meilin Tian†, Yangping Li†, Jing Jing†, Chuang Mu, Hengde Li, Huixia Du, Jinzhuang Dou, Junxia Mao, Xue Li, Wenqian Jiao, Yangfan Wang, Xiaoli Hu, Shi Wang*, Ruijia Wang* and Zhenmin Bao

Supplementary Figure F1. Illustration of "dominant tag", "dominant marker", "codominant tag" and "codominant marker".

Supplementary Figure F2. Illustration of female-specific genetic linkage map of *A. japonicus*.

Supplementary Figure F3. Illustration of male-specific genetic linkage map of *A. japonicus*.

Supplementary Figure F4. Illustration for possible pairing of two types of selective adaptors.

Supplementary Table T1:

Information for shared markers in each linkage group of sex-specific maps and consensus map.

Supplementary Table T2:

Genotyping consistency of parents and 10 progenies.

Additional file A1:

Number of raw reads, reads amount after reads filtering and reads mapping for parents and progenies.

Additional file A2:

Information of mapped markers for sex-specific maps and consensus map in A. japonicas.

Additional file A3:

Information of the result of association analysis on body weight of *A. japonicas* including original P-value and adjusted ones.

Construction of a High-Density Genetic Map and Quantitative Trait Locus Mapping in Sea Cucumber, *Apostichopus japonicus*

Meilin Tian[†], Yangping Li[†], Jing Jing[†], Chuang Mu, Huixia Du, Jinzhuang Dou, Junxia Mao, Xue Li, Wenqian Jiao, Yangfan Wang, Xiaoli Hu, Shi Wang[*], Ruijia Wang[*] and Zhenmin Bao

Supplementary Figure F1:

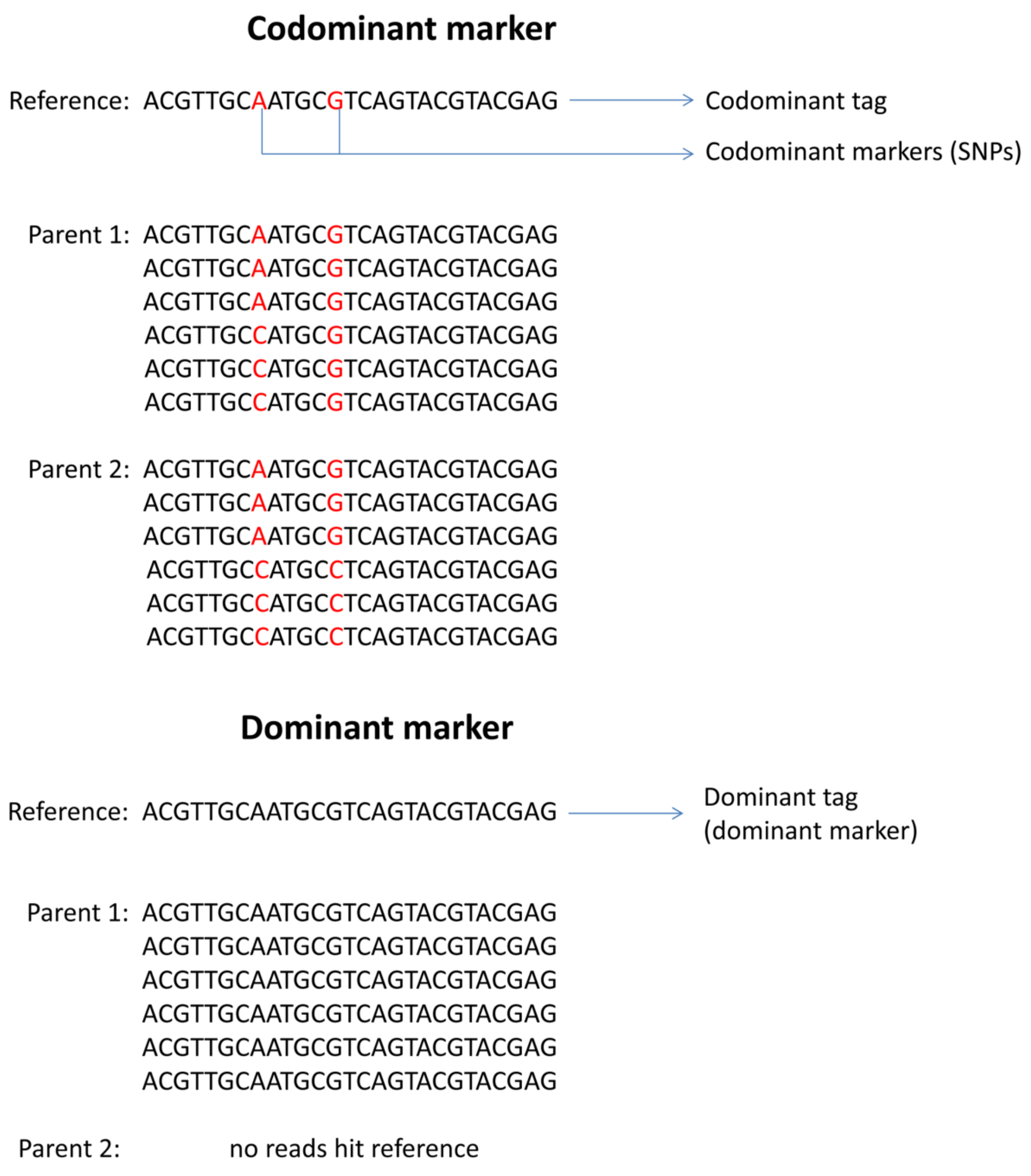

Supplementary Figure F2:

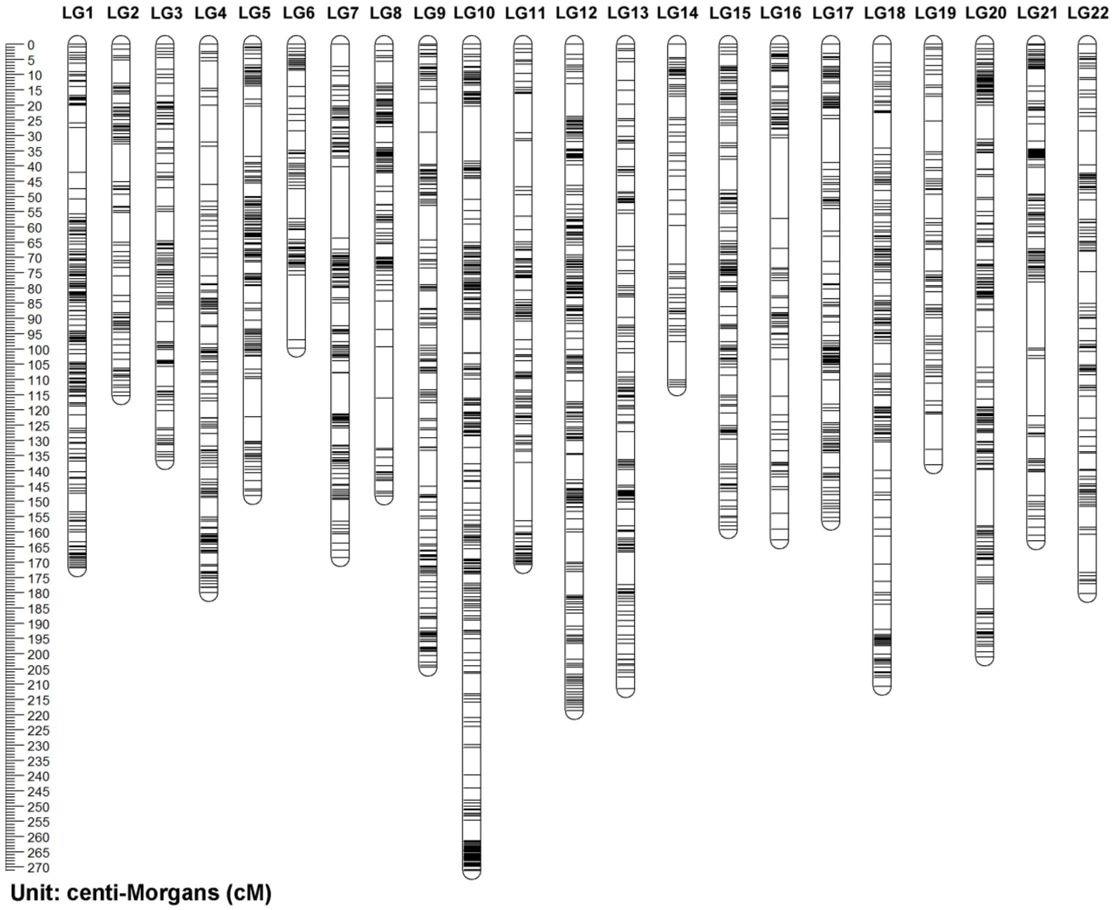

Supplementary Figure F3:

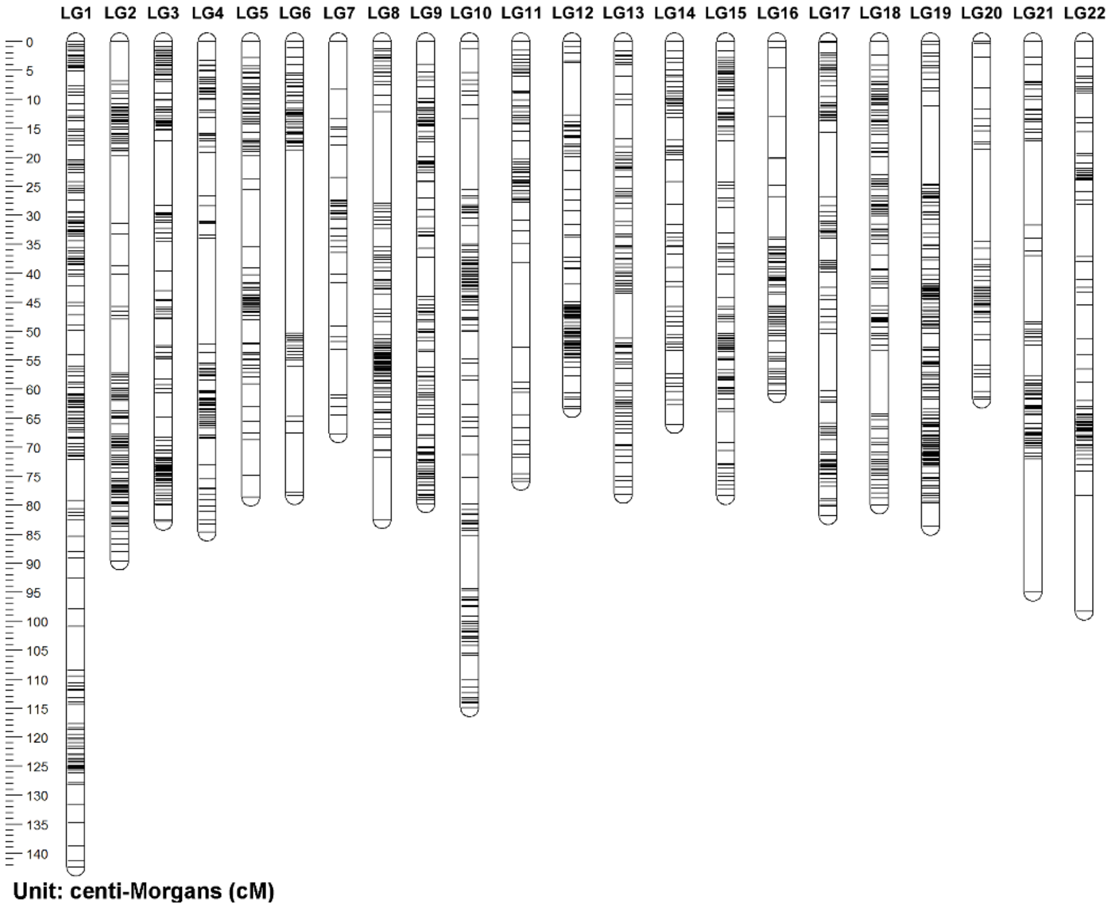

Supplementary Figure F4:

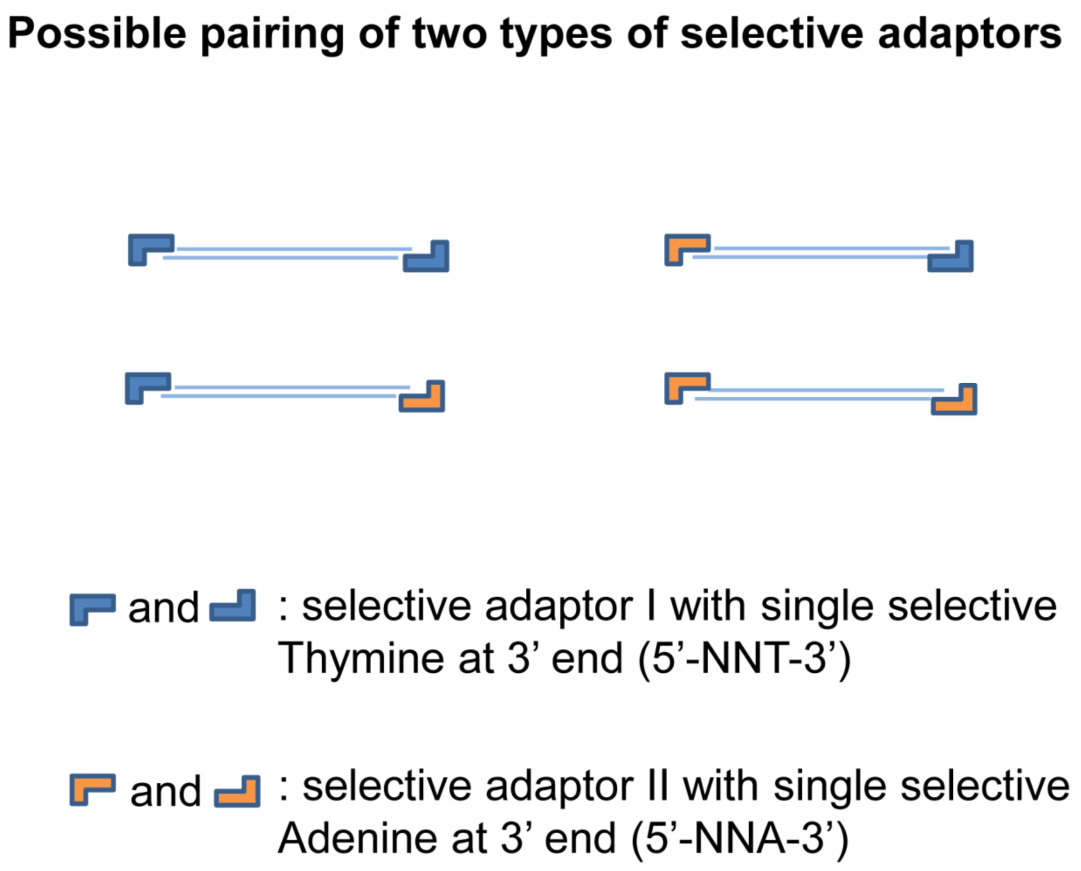

Supplementary Table T1: Information for shared markers in each linkage group of sex-specific maps and consensus map

	Female map				Male map				Consensus map			F:M ratio of
**Linkage group**	**Mapped markers**	**Distinct positions**	**Marker interval** (cM)	**Average recombination rate**	**Mapped markers**	**Distinct position**	**Marker interval** (cM)	**Average recombination rate**	**Distinct position**	**Marker interval (cM)**	**Average recombination rate**	**average recombination rate**
**1**	31	27	5.07	0.049	24	23	6.01	0.058	35	4.64	0.045	0.83
**2**	3	3	0.1	0.103	6	6	12.38	0.112	8	8.84	0.086	0.92
**3**	20	18	0.05	0.055	17	17	4.93	0.047	26	4.02	0.038	1.17
**4**	14	14	0.09	0.092	9	9	8.04	0.073	16	8.30	0.080	1.26
**5**	12	12	10.96	0.084	5	5	13.41	0.125	14	8.44	0.074	0.67
**6**	9	8	8.11	0.078	7	7	7.59	0.069	12	5.48	0.054	1.12
**7**	10	9	17.76	0.142	9	8	8.46	0.077	11	14.89	0.126	1.84
**8**	12	11	11.81	0.087	13	12	4.17	0.020	14	9.17	0.068	4.32
**9**	11	11	18.46	0.152	8	7	9.35	0.087	14	14.04	0.126	1.75
**10**	23	23	11.8	0.098	19	19	5.55	0.054	29	8.72	0.081	1.82
**11**	19	19	8.86	0.085	10	10	6.66	0.064	21	7.40	0.071	1.32
**12**	17	17	11.03	0.103	13	13	3.97	0.040	19	9.31	0.083	2.61
**13**	24	22	7.69	0.074	15	14	5.08	0.050	24	6.41	0.062	1.48
**14**	8	8	6.08	0.059	10	9	5.69	0.056	12	4.70	0.047	1.06
**15**	20	19	7.76	0.074	18	17	4.33	0.043	24	5.68	0.055	1.73
**16**	7	7	26.48	0.237	6	6	11.94	0.115	8	18.94	0.178	2.06
**17**	6	6	22.09	0.183	15	14	5.17	0.050	16	7.36	0.072	3.64
**18**	11	11	18.92	0.158	7	7	11.91	0.114	12	15.56	0.141	1.38
**19**	10	9	13.18	0.108	15	15	4.8	0.047	19	5.49	0.052	2.31
**20**	16	14	12.54	0.099	8	7	7.92	0.076	15	11.49	0.098	1.31
**21**	7	6	11.28	0.093	5	5	4.96	0.049	8	7.57	0.072	1.88
**22**	16	15	9.53	0.081	10	10	8.21	0.072	16	8.59	0.076	1.12
**Total**	306	289	10.89	0.104	249	240	7.3	0.068	373	8.86	0.081	1.53

Supplementary Table T2: Genotyping consistency of parents and 10 progenies

Resequencing individuals	Co-efficiency (%)
**Progeny 1**	93.39
**Progeny 2**	94.41
**Progeny 3**	96.96
**Progeny 4**	95.42
**Progeny 5**	92.86
**Progeny 6**	95.03
**Progeny 7**	92.79
**Progeny 8**	91.23
**Progeny 9**	95.61
**Progeny 10**	92.92
**Father**	96.90
**Mother**	95.00
**Average of 10 progenies**	94.06
**Average of all resequencing individuals**	94.38
